# The spectrum of mitochondrial DNA (mtDNA) mutations in pediatric CNS tumors

**DOI:** 10.1093/noajnl/vdab074

**Published:** 2021-06-02

**Authors:** Kristiyana Kaneva, Katrina O’Halloran, Petr Triska, Xiyu Liu, Daria Merkurjev, Moiz Bootwalla, Alex Ryutov, Jennifer A Cotter, Dejerianne Ostrow, Jaclyn A Biegel, Xiaowu Gai

**Affiliations:** 1 Division of Hematology-Oncology, Neuro-Oncology & Stem Cell Transplantation, Ann & Robert H. Lurie Children’s Hospital of Chicago, Chicago, Illinois, USA; 2 Department of Pathology, Children’s Hospital Los Angeles, Los Angeles, California, USA; 3 Department of Paediatric Haematology and Oncology, Second Faculty of Medicine of Charles University, Prague, Czech Republic

**Keywords:** brain tumors, CBTN, CBTTC, CNS tumors, mitochondrial DNA, pediatric

## Abstract

**Background:**

We previously established the landscape of mitochondrial DNA (mtDNA) mutations in 23 subtypes of pediatric malignancies, characterized mtDNA mutation profiles among these subtypes, and provided statistically significant evidence for a contributory role of mtDNA mutations to pediatric malignancies.

**Methods:**

To further delineate the spectrum of mtDNA mutations in pediatric central nervous system (CNS) tumors, we analyzed 545 tumor-normal paired whole-genome sequencing datasets from the Children’s Brain Tumor Tissue Consortium.

**Results:**

Germline mtDNA variants were used to determine the haplogroup, and maternal ancestry, which was not significantly different among tumor types. Among 166 (30.5%) tumors we detected 220 somatic mtDNA mutations, primarily missense mutations (36.8%), as well as 22 loss-of-function mutations. Different pediatric CNS tumor subtypes had distinct mtDNA mutation profiles. The number of mtDNA mutations per tumor ranged from 0.20 (dysembryoplastic neuroepithelial tumor [DNET]) to 0.75 (meningiomas). The average heteroplasmy was 10.7%, ranging from 4.6% in atypical teratoid/rhabdoid tumor (AT/RT) to 26% in diffuse intrinsic pontine glioma. High-grade gliomas had a significant higher number of mtDNA mutations per sample than low-grade gliomas (0.6 vs 0.27) (*P* = .004), with almost twice as many missense mtDNA mutations per sample (0.24 vs 0.11), and higher average heteroplasmy levels (16% vs 10%). Recurrent mtDNA mutations may represent hotspots which may serve as biologic markers of disease.

**Conclusions:**

Our findings demonstrate varying contributions of mtDNA mutations in different subtypes of CNS tumors. Sequencing the mtDNA genome may ultimately be used to characterize CNS tumors at diagnosis and monitor disease progression.

**Key Points**

- Childhood brain tumors demonstrate a spectrum of somatic mtDNA mutations.
- High-grade gliomas harbor a statistically significantly higher number of mtDNA mutations per sample than low-grade gliomas at higher average heteroplasmy levels.
- Loss-of-function mutations, including multiple recurrent alterations, cluster in complex I genes.

**Importance of the Study**

This is the most comprehensive germline and tumor mtDNA study to date that focuses exclusively on pediatric CNS tumors.

Metabolism has been thought to play an important role in cancer for nearly a century.^1,2^ Otto Warburg first described what is referred to as the Warburg effect in 1927 when he observed that cancer cells preferentially undergo glycolysis even in the presence of oxygen, erroneously concluding that mitochondria were not used by cancer cells.^3^ The role of mitochondria in cancer is in fact much more complex and dynamic and still poorly understood. It has been known for some time that mitochondrial biogenesis and turnover, the signaling mechanism within mitochondria in response to oxidative stress, fission and fusion dynamics, metabolism, and bioenergetics all play a role in tumorigenesis, tumor metastasis, and cancer progression.^3–5^ The recently discovered mitochondrial unfolded protein response (UPR^mt^) pathway, for example, is thought to activate the transcription of nuclear-encoded mitochondrial proteins that rescue damaged mitochondria as a mechanism that prolongs cancer cell survival.^6,7^ The structure and function of mitochondria are programmed by both nuclear-encoded genes and maternally inherited mitochondrial DNA (mtDNA).^8^ Each cell contains 1 nuclear genome but as many as hundreds to thousands of copies of the mtDNA genome, which results in extraordinary genomic heterogeneity. Germline and somatic variation or mutations in the mtDNA are typically present in a fraction of mutant mtDNA genomes compared to the total mtDNA genomes in a tissue or cell, referred to as heteroplasmy.^9^ Conceivably, mtDNA heteroplasmy could be a fitting yet elegant mechanism to fine tune and to modulate inter- and intratumoral heterogeneity.

Previous studies, focusing primarily on adult cancers, identified mtDNA mutations in a variety of human malignancies, but until recently they were largely dismissed as passenger events.^4,10^ Mok et al. showed that editing *MT-ND4* using a bacterial cytidine deaminase toxin resulted in lower rates of oxidative phosphorylation and decreased basal and uncoupled respiratory rates consistent with complex I disruption.^11^ In a pan-cancer analysis of data from The Cancer Genome Atlas project, Yuan et al. provided compelling evidence for the oncogenic effects of mtDNA mutations in tumors, and identified truncating mtDNA mutations in colorectal, kidney, and thyroid tumors.^12^ Findings from the Yuan et al. study are in agreement with our previous pan-cancer study of pediatric malignancies.^13^ We originally described the landscape of germline and somatic mtDNA mutations in a large heterogeneous collection of 616 pediatric malignancies which included 142 central nervous system (CNS) tumors.^13^ We established varying mtDNA mutation profiles across 23 subgroups of hematologic malignancies and solid tumors and demonstrated statistically significant associations of mtDNA mutations, especially loss-of-function (truncating) mutations in different tumor subtypes, including CNS tumors.^13^

Pediatric brain tumors are the most common solid malignancy in children and the second cause of cancer-related morbidity and mortality in this population.^14^ Previously, a number of small-scale pediatric studies reported mtDNA mutations in CNS tumors including medulloblastoma (MB) and other pediatric brain tumors.^15–17^

In this study, we focused exclusively on pediatric CNS tumors and mined 545 paired tumor-normal whole-genome sequencing (WGS) datasets obtained through the Children’s Brain Tumor Network (CBTN), formerly the Children’s Brain Tumor Tissue Consortium (CBTTC) (Supplementary Table 1). CBTN is a collaborative effort dedicated to compiling somatic and germline genomic data from pediatric brain and spinal cord tumors. The CBTN first assembled data from 17 participating institutions across the United States as well as internationally. Due to the large sample size and comprehensive data collection, the CBTN database has been used for several studies focused on genomic patterns in different pediatric CNS tumors including, for example, a study that discerned the distinct genomic alterations in pediatric high-grade gliomas (HGGs).^18^ The CBTN data include WGS datasets from tissue samples as well as matched blood for germline analysis. The datasets enabled us to determine the haplogroup for each patient, and thus maternal ancestry, and to characterize the spectrum of somatic mtDNA mutations in pediatric CNS tumors, providing further evidence that mtDNA mutations contribute to tumor development and/or progression.

## Materials and Methods

### Ethics Statement

The pediatric brain and spinal cord tumor patients studied here were originally consented to the CBTTC study. We were granted access to the completely deidentified CBTTC datasets through a data use agreement between Children’s Hospital Los Angeles and the CBTTC. The CBTTC/CBTN data analyzed in this study included WGS tumor and matched normal data from 17 different institutions (https://cbtn.org/).

### mtDNA Mutation and Variant Detection

We extracted mtDNA reads from paired tumor-normal WGS data and aligned them to GRCh37 using NovoAlign (Novocraft Technologies Sdn Bhd) with default parameters. DNA somatic and germline calls were made with the Edico DRAGEN Genome genome pipeline (version 2.3.1) (Illumina Inc.) using default settings and filters and further filtered by our LUBA variant caller.^19^ The custom LUBA variant caller generates a pile-up file from aligned reads, and base counts are adjusted depending on base qualities. Benjamini–Hochberg correction was applied to control the false discovery rate. Calls were then made for loci with sufficient coverage. The LUBA variant caller was run with the same parameters as VarScan2 (stringent strand-bias filter, at least 2% alternative allele frequency, and minimum 100× coverage) and was used to confirm calls made by VarScan2.^20^

For somatic mtDNA mutations, we filtered out frequently recurrent calls (>10%) and also the common germline variants based on the publicly available database of mitochondrial variants MitoMap (https://mitomap.org).^21^ Haplogroup calls were made by Phy-Mer using BAM files without the need for variant calling, and the most likely haplogroup was chosen for the final haplogroup.^22^ Variants were then plotted using the Triska Mito app (https://triska-mitoapp.herokuapp.com/). The average number of somatic mutations per sample by tumor type and the average variant allele frequency (VAF) by CNS tumor diagnosis and the 95% confidence interval were calculated by the *t*-test function in R and plotted by the ggplot2 package (https://www.rdocumentation.org/packages/ggplot2/versions/3.3.2). The composition of somatic mutations by tumor subtype was plotted by the circlize package (https://www.rdocumentation.org/packages/circlize/versions/0.4.10). For the profile of mtDNA mutations and variants, the composition and the heteroplasmy level of the somatic mutations were plotted with the ggplot2 package in a stack bar chart. The Fisher’s exact test was used to determine whether there was a statistical difference between 2 groups, using the fisher.test function in R.

## Results

### Study Subjects and mtDNA Genome Sequencing

We analyzed data from 545 matched tumor (somatic) and normal (germline) WGS datasets across 25 different pediatric CNS tumor histologic subtypes (Supplementary Figure 1; Supplementary Table 2). The most prevalent subgroup was low-grade glioma (LGG) not otherwise specified (NOS) (30%), followed by ependymoma (EPN) and MB each 11%, and HGG (9%) (Supplementary Table 3). Of all 545 participant cases, 303 (56%) were male and 242 (44%) were female. The demographic data on race and ethnicity were limited in this database and was likely confounded by a bias in self-reporting (Supplementary Table 2). With regard to race, 344 (63%) of the participants were white, 43 (7.9%) identified as black or African American, 13 (2.4%) were Asian, 6 (1.1%) were American Indian or Alaskan native, 1 (0.2%) Native Hawaiian or other Pacific Islander, 2 (0.4%) reported more than 1 race, for 2 (0.4%) there were no data, and 134 (24.6%) chose not to report their race. The ethnicity analysis revealed that 425 (78%) of participants were non-Hispanic, 57 (10.4%) were Hispanic or Latino, 61 (11.2%) chose not to report their ethnicity, and for 2 (0.4%) there were no data available.

Due to the high copy number of mtDNA genomes, the standard genome coverage provides much higher coverage (>1000×) for the mtDNA genome in both the tumor samples as well as matched blood compared to the nuclear genome (Supplementary Table 4). The high mtDNA coverage generated through next generation sequencing allowed us to analyze low-level heteroplasmy or VAF while minimizing potential non-mtDNA genome reads derived from contaminating nuclear mtDNA transcripts (“NuMTs”).^23^

### Tumor-Only mtDNA Mutation Profiles

**Figure 1.**
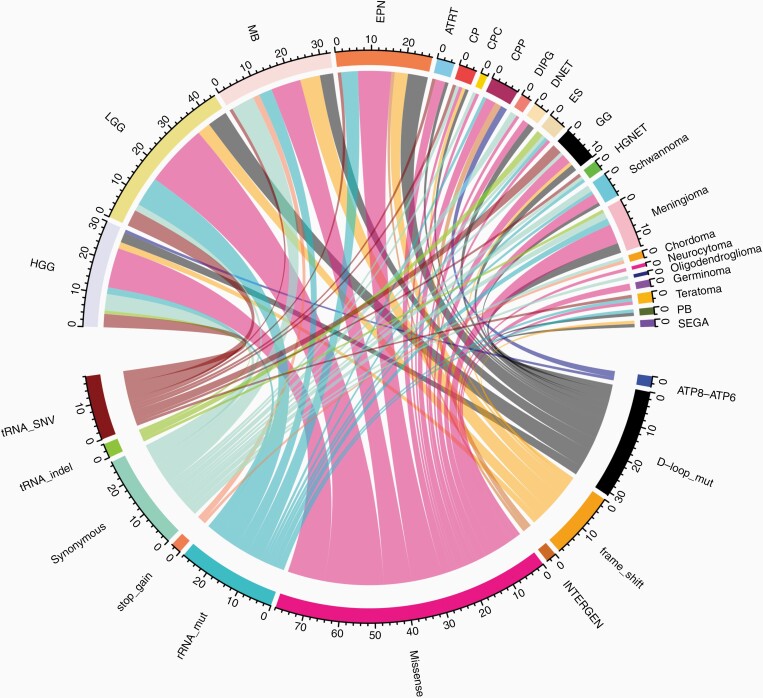
Composition of types of somatic mtDNA variants (bottom) in each CNS tumor subtype (top). CNS, central nervous system; mtDNA, mitochondrial DNA.

The mtDNA variants were called in each tumor sample, as well as the matched normal sample (Supplementary Table 5). Germline variants or the variants detected in the normal sample were subtracted from the mtDNA variants found in the tumor sample, and this left 200 somatic heteroplasmic mtDNA mutations in 166 patients (Supplementary Table 6). We excluded 5 patients with multiple diagnoses from categorical analysis and these samples were analyzed separately as detailed below. For patients with multiple biospecimens, findings from the initial specimen were included in the categorical analysis and recurrent/progressive specimens were analyzed separately. A total of 214 tumor-only mtDNA mutations were detected in 160 (29.6%) of the remaining 540 CNS tumors (Supplementary Table 6; Figure 1). Most frequently observed were missense mutations (80, 36.4%). We also detected 22 loss-of-function (LoF) mutations, which included both the nonsense and frameshift mutations. *D-loop* mutations were noted most frequently including 35 heteroplasmic alterations, followed by *MT-CYTB* and *MT-ND5* with 22 and 21 heteroplasmic mutations, respectively. Genes with 10 or more heteroplasmic mutations across tumor types included *MT-RNR2* (18), *MT-COX1* (16), *MT-ND2* (15), *MT-ND1* (12), *MT-ND4* (11), and *MT-RNR1* (10).

### mtDNA Mutations by CNS Tumor Subtype (*n* > 15)

For categorical analysis, we focused on the tumor subtypes with the highest number of patients (LGG), tumors with greater than 50 patients (EPN, MB, and HGG), and tumor types with at least 15 patients which included atypical teratoid/rhabdoid tumor (AT/RT), DNET, meningioma, craniopharyngioma, and ganglioglioma.

**Figure 2.**
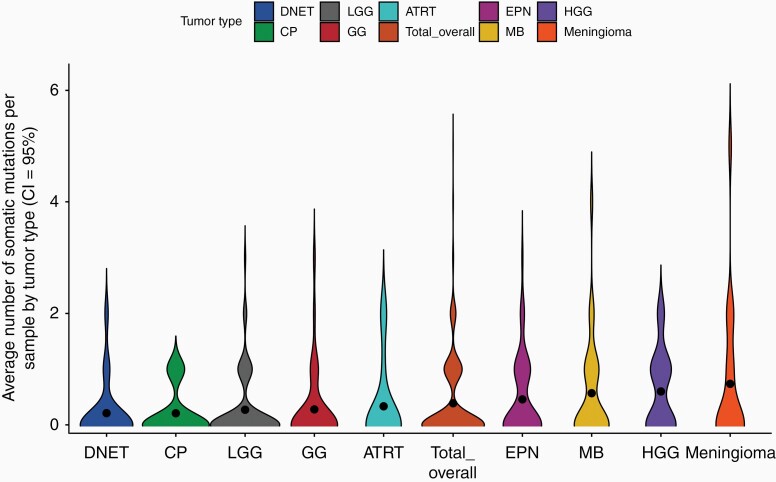
Number of mutations per sample by diagnosis.

The mean number of tumor-only mtDNA mutations seen across all subtypes with at least 15 cases was 0.41, ranging from 0.20 in DNET to 0.74 in meningioma (Figure 2). Tumor subtypes with at least 15 samples each were largely grouped into 2 categories: tumor subtypes with higher number of mtDNA mutations per sample, and those with lower number of mtDNA mutations per sample. Tumor subtypes with lower numbers of mtDNA mutations per sample included DNET, craniopharyngioma, ganglioglioma, and LGG. All of these tumors are also low grade and clinically less aggressive lesions. This is in notable contrast to tumors found to have a higher number of mtDNA mutations per sample, including a AT/RT, MB, EPN, and HGG which are generally high-grade lesions and represent clinically aggressive tumors. Specifically, HGGs had a higher number of mtDNA mutations per sample than LGGs (0.6 vs 0.27) (*P* = .003521), with almost twice as many missense mtDNA mutations per sample (0.24 vs 0.11), although the difference is not statistically significant (*P* = .08723). This suggests that mtDNA mutations may play a more important role in HGGs compared to LGGs.

### Tumor Types With a Low Number of mtDNA Mutations per Sample

LGGs represent the most common tumor diagnosis in this dataset (*n* = 166) which demonstrated a mean number of mutations per sample of 0.27 with an average heteroplasmy level of 0.1. Missense mutations were the most abundant, representing 19 of 45 total tumor-only mutations that primarily cluster in *MT-CYTB* (7), *MT-ND5* (4), and *MT-ND2* (3). Gangliogliomas (*n* = 36) harbored only 10 heteroplasmic mutations with 0.28 mean mutations per sample and an average heteroplasmy of 0.09. A total of 3 missense mutations were identified including a missense mutation in *MT-CYTB*, *MT-ND2*, and *MT-ND4*. Craniopharyngioma (*n* = 24) similarly showed a paucity of mtDNA alterations with just 5 heteroplasmic mutations identified, one of which was a missense mutation in *MT-CO1*. DNETs (*n* = 19) carried a total of 4 heteroplasmic mutations. The average number of mutations per sample was lowest in DNETs at 0.2 with a low heteroplasmy level of 0.07. Two missense mutations were identified in DNETs, 1 in *MT-CO1* and 1 in the *MT-ND3* gene.

### Tumor Types With Higher Numbers of mtDNA Mutations per Sample

HGGs (*n* = 50) had a higher number of mtDNA mutations per sample than the LGGs. Overall, 0.6 mutations per sample are noted in HGGs (second only to meningioma with 0.74) and the average VAF was 0.16. Again, a predominance of heteroplasmic missense mutations was noted, representing 12 of 30 (40%) tumor-only mutations. Mutations appear to cluster in *MT-COX1* (2), *MT-CYTB* (2), and *MT-ND2* (2) in HGGs. Additionally, 2 frameshift mutations were noted in *MT-COX1* and *MT-ND4*, respectively. EPNs (*n* = 59) were also noted to have a higher number of mtDNA mutation per sample with 27 total heteroplasmic mutations and 0.46 mutations per sample. EPNs harbored 10 missense mutations in total with multiple mutations in *MT-CO3* (3), *MT-CO1* (2), *MT-ND1* (2) as well as 4 frameshift mutations in *MT-ND1*, *MT-ND2*, *MT-ND3*, and *MT-ND5* and 6 *rRNA* mutations. Similarly, MBs (*n* = 58) were found to harbor 33 heteroplasmic mutations overall with a mean number of mutations per sample of 0.57 and a heteroplasmy level of 0.17. Of all heteroplasmic mutations, 9 represented missense mutations in *MT-CYTB* (4), *MT-ND1* (2), *MT-ND2* (2) and 6 were frameshift mutations in *MT-ND5* (3), *MT-ND3*, *MT-ND4*, and *MT-ND6*. ATRTs (*n* = 15) contained a total of 0.33 mutations per sample with a low average heteroplasmy of 0.04. Missense mutations represented 3 of the 5 heteroplasmic changes in AT/RTs and were found in *MT-ND2*, *MT-CO3*, and *MT-CYTB*.

Meningiomas (*n* = 19) had 14 heteroplasmic mutations and thus the highest average number of mutations per sample, 0.74, and an average heteroplasmy of 0.12. Six missense mutations were noted in *MT-CO1* (2), *MT-CYTB* (1), *MT-ND3* (1), *MT-ND4L* (1), and *MT-ND5* (1).

**Figure 3.**
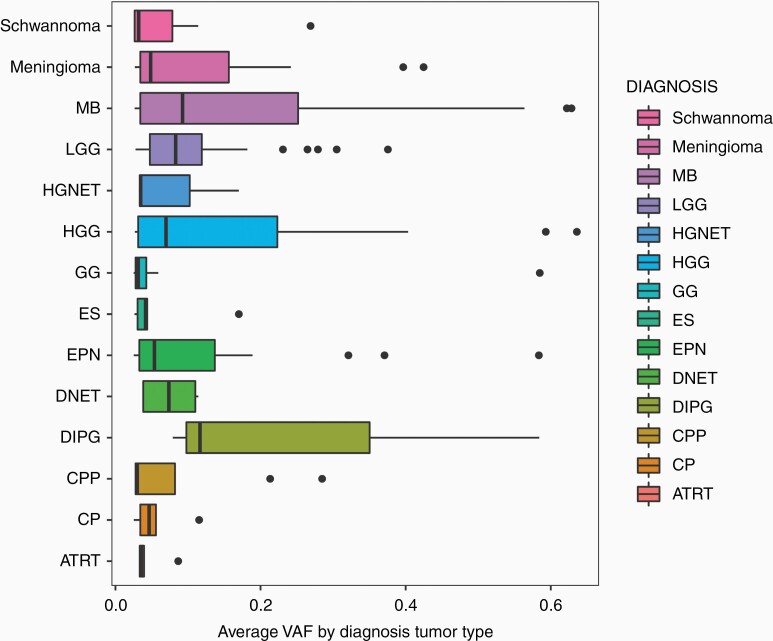
Variant allele frequency (heteroplasmy) by diagnosis.

As noted above, the VAF or heteroplasmy level varied by tumor type. The overall mean VAF was 0.107, indicating that mitochondrial variant alleles were detected at an average of just over 10% of reads aligned at the variant position. The lowest variant allele frequencies were seen in AT/RT with an average VAF of 0.04 and the highest were seen in diffuse intrinsic pontine glioma (DIPG) with an average VAF of 0.26 (Figure 3).

### Diagnoses With Small Sample Number (*n* < 15)

Diagnostic categories with fewer than 15 samples each included choroid plexus papilloma (CPP) (*n* = 14), subependymal giant cell astrocytoma (SEGA) (*n* = 14), schwannoma (*n* = 11), teratoma (*n* = 9), high-grade neuroepithelial tumors (HGNET) (*n* = 8), Ewing sarcoma (*n* = 6), germinoma (*n* = 4), pineoblastoma (*n* = 4), DIPG (*n* = 4), choroid plexus carcinoma (CPC) (*n* = 3), neurocytoma (*n* = 3), chordoma (*n* = 2), and oligodendroglioma (*n* = 2). In addition, we reviewed 10 biopsy samples from gliosis, a nonspecific reactive change of glial cells secondary to CNS damage.^24^ Gliosis changes involve cellular proliferation or hypertrophy but are not considered neoplastic and were excluded from our downstream categorical analyses. Five heteroplasmic mutations were identified in CPPs with 1 missense mutation in *MT-CO1*. Only 2 mutations were identified in SEGAs including a frameshift *MT-ND1* mutation. Seven heteroplasmic mutations were identified across schwannoma samples with 3 missense mutations identified in *MT-CO3*, *MT-ND4*, and *MT-CYTB*. Three mutations were identified in teratomas including a missense mutation in *MT-ND5.* Five mutations were noted in Ewing sarcoma samples with 1 missense mutation in *MT-ND5*. Germinomas harbored 2 heteroplasmic mutations, both missense in *MT-ND5* and *MT-CYTB*, respectively. A single *MT-ND6* missense mutation was identified in DIPG samples. Across CPCs, 1 missense *MT-CO1* mutation was identified. One missense *MT-ND5* mutation was seen in a neurocytoma. No missense mutations were identified in HGNET, pineoblastoma, chordomas, and oligodendrogliomas. For comparison, 3 heteroplasmic mutations were identified in a total 3 of 10 gliosis samples and none of those were missense (1 rRNA mutation m.2140G>A at 6.9% heteroplasmy, 1 ND6 synonymous mutation m.14569G>A at 12% heteroplasmy, and 1 COX1 synonymous mutation m.6158A>G at 4.7% heteroplasmy).

### Clustering of Loss-of-Function Somatic mtDNA Mutations

As suggested in our previous study and by Yuan et al., truncating or loss-of-function (LoF) mtDNA mutations are more likely to be oncogenic.^12,13^ In the CBTN CNS tumor dataset studied here we identified a total of 22 LoF mtDNA mutations which included both frameshift and stop-gain mutations (Table 1). The majority of these LoF mutations were located in mitochondrial complex I genes, with the most (4 each) in *MT-ND5* and *MT-ND4*. No LoF mutations were observed in *MT-CO3* or *MT-ATP8*. The most mtDNA LoF mutations were present in MBs with 8 LoF mutations among 58 MBs total (0.14 per sample). A single MB had 2 LoF mutations, 1 in *MT-ND2* and 1 in *MT-ND5*. Thus, 7 of the 58 (12%) of the MBs had at least 1 mtDNA LoF mutation. We observed an interesting mtDNA mutation pattern in chordomas. The same chordoma patient had 3 LoF mutations, 2 in *MT-ND4* and 1 in *MT-CO2*. Additionally, 2 different chordoma cases harbored the same stop-gain mutation in *MT-ND4*, m.10971G>A.

**Table 1.** Clustering of Loss-of-Function Mutations

Mitochondrial Region	Gene	Number of LoF Mutations	CNS Tumor Subtype
Complex I	*MT-ND6*	1	MB
	*MT-ND5*	4	MB (3), EPN
	*MT-ND4*	4	Chordoma (1), HGG, LGG, MB
	*MT-ND3*	2	EPN, MB
	*MT-ND2*	3	LGG, EPN, MB
	*MT-ND1*	3	EPN, LGG, SEGA
Complex III	*MT-CYB*	1	MB
Complex IV	*MT-CO3*	0	—
	*MT-CO2*	1	CP
	*MT-CO1*	2	LGG, HGG
ATP synthase	*MT-ATP8*	0	—
	*MT-ATP6*	1	GG

CNS, central nervous system; CP, craniopharyngioma; EPN, ependymoma; GG, ganglioglioma; HGG, high-grade glioma; LGG, low-grade glioma; MB, medulloblastoma; SEGA, subependymal giant cell astrocytoma.

Notably, multiple recurrent LoF mutations were seen in multiple tumors of different subtypes (Supplementary Table 7). A recurrent *MT-ND3* LoF mutation, m.10191TC>T, was observed in 1 MB and 1 EPN. The same LoF mutation in *MT-ND4*, m.10946A>AC was observed in a LGG and a HGG. Another recurrent LoF mutation in *MT-ND4*, m.11031GA>G was seen in a chordoma case and MB case. Lastly, m.12417CA>C, a LoF mutation in *MT-ND5* was seen in a HGG, MB, and craniopharyngioma.

### Hypermutated Samples

Assessing nuclear DNA mutations was beyond the scope of this study. However, it is known that mutations in mismatch repair or other DNA repair pathways loci often lead to a hypermutation profile, characteristic of a subset of brain tumors, primarily HGGs. We therefore sought to determine if the mtDNA genome was also hypermutated in these tumors, which would suggest shared or overlapping underlying mechanisms causing both nuclear DNA and mtDNA mutations. Ijaz et al. analyzed nuclear mutations in pediatric HGG from the CBTN dataset.^18^ and identified 7 HGGs with high or ultra-high mutational burden (high = 10–100 mutations/Mb, ultra high ≥100 mutations/Mb). Five of those same that were also included in this study were evaluated. Three of these samples demonstrated missense mutations in *MT-ND2*, *MT-CYTB*, and *MT-CO1*, respectively. One sample contained no heteroplasmic mitochondrial mutations. The final sample showed a *D-loop* mutation and an intergenic mutation. Aside from these 5 samples, the highest number of mutations in a single sample across all diagnoses in our dataset was in a meningioma with 5 heteroplasmic mutations (sample ID BS_2W200YK5) which showed missense mutations in *MT-CO1*, *MT-ND3*, and *MT-ND4L* as well as an *RNR1* mutation and a *D-loop* mutation. This suggests that mtDNA hypermutation in pediatric brain tumors is rare and hypermutation in the nuclear DNA genome does not correlate with hypermutation in the mtDNA genome.

### Cases With Multiple Tumor Specimens

WGS data were available from multiple tumor biospecimens from 64 patients including 13 patients with HGG, 10 with LGG, 2 with LGG at initial biopsy followed by HGG at recurrence, as well as 9 patients with EPN and 4 with MB. There were also 12 cases with multiple biospecimens from the same day.

Biospecimens from the same day appeared to show both identical mtDNA mutations across same day specimens, but also differences from 1 specimen to the next. For example, 2 HGG specimens taken from the same day showed the identical *MT-ND6* m.14553C>T missense mutation, however only one of the same day specimens was shown to have an *MT-* m.4219G>A *ND1* missense mutation (biospecimens BS_68TZMZH1, BS_AFBPM6CN). These findings suggest that there is intratumoral heterogeneity, similar to nuclear mutations.

In patients with multiple biospecimens from different timepoints (eg, a tissue biopsy from the initial diagnosis followed by a specimen from progression or recurrence), we found shared or persistent mutations, as well as mutations which were later lost or gained. For example, 1 patient had multiple biospecimens available from initial LGG diagnosis, LGG progression, first relapse with HGG and second relapse with HGG (ID PT_2WVW55DA). This patient’s initial diagnostic specimen showed an *MT-ND4* stop-gain mutation m.11922G>A which was not detected at the time of LGG progression, however, a *MT-COX2* missense m.7847G>A mutation was present. At the time of relapse with HGG both mutations had been lost and no additional somatic mutations were identified. Conversely, another patient (ID PT_HT4HJXY6) with an initial diagnosis of MB and no mtDNA mutation was later diagnosed with meningioma which had an m.11682G>A *MT-ND4* missense mutation.

Overall, interestingly, HGGs with multiple specimens showed the highest number of mtDNA mutations throughout multiple samples. Furthermore, a majority (7 of 11) of these mutations were in complex I genes, including 2 *MT-ND1*, 2 *MT-ND5*, and 2 *MT-ND6* mutations in addition to a *MT-ND2* mutation. Similar results were seen in LGG with multiple mutations in complex I genes, including *MT-ND2*, *MT-ND4*, and *MT-ND5* as well as in *MT-COX1* and *MT-COX2* mutations.

A patient with a diagnosis of chordoma (ID PT_HFQNKP5X) had data from 4 biospecimens with 1 from the time of initial diagnosis and 3 at recurrence/progression. The initial specimen showed a synonymous *MT-COX2* mutation (m.8251G>A) and no other mtDNA mutations. Of the 3 samples from progression/relapse: 1 specimen showed a missense *MT-ND2* m.5338T>C mutation and an *MT-TRNA* m.10054G>A mutation; the second showed a *MT-COX3* stop-gain mutation at m.9687C>T and a *MT-ND4* stop-gain m.10971G>A mutation as well as a *MT-ND4* frameshift m.11031GA>G mutation; the third harbored a missense *MT-ND2* m.5338T>C mutation.

### Mitochondrial Haplogroup Analysis

**Figure 4.**
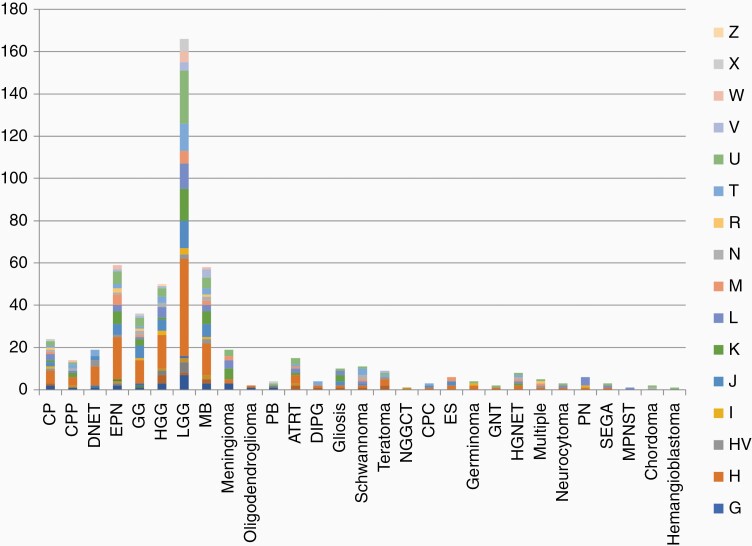
Composition of mitochondrial major haplogroups in patients of each tumor type.

In order to determine if maternal ancestry was associated with tumor subtype in the CBTN dataset, the mitochondrial haplogroup for each patient was also determined.^22^ (Supplementary Table 8). Macrohaplogroups included R, M, N, L, and major haplogroups included A, B, C, D, E, F, G, H, HV, I, J, K, L, M, N, R, T, U, V, W, X, and Z (Supplementary Figure 2). In contrast to the self-reported race and ethnicity data outlined above, mitochondrial haplogroup analysis showed that 389 (71%) patients belonged to macrohaplogroup “R,” 66 (12%) clustered in group “N,” 45 (8%) in group “M,” and 45 (8%) in group “L.” Major haplogroup analysis showed the largest number of patients belonged to haplogroup “H” with 152 (28%) patients, followed by 65 (10%) patients in haplogroup “U,” 45 (8%) each in groups “J” and “L” and fewer than 45 patients in the remaining haplogroups (Figure 4). The tumor diagnoses with the largest number of subjects including LGG, HGG, MB, and EPN each had the Eurasia-centric H haplogroup represented most frequently. This observation likely reflects a population bias given the preponderance of the H haplogroup overall in this dataset. Conversely, subjects from the H haplogroup manifested 24 different tumor types and within each tumor diagnosis there were numerous haplogroups represented without any specific haplogroup-tumor correlations (Figure 4).

## Discussion

### Tumor-Only mtDNA Mutations Across CNS Tumors

**Figure 5.**
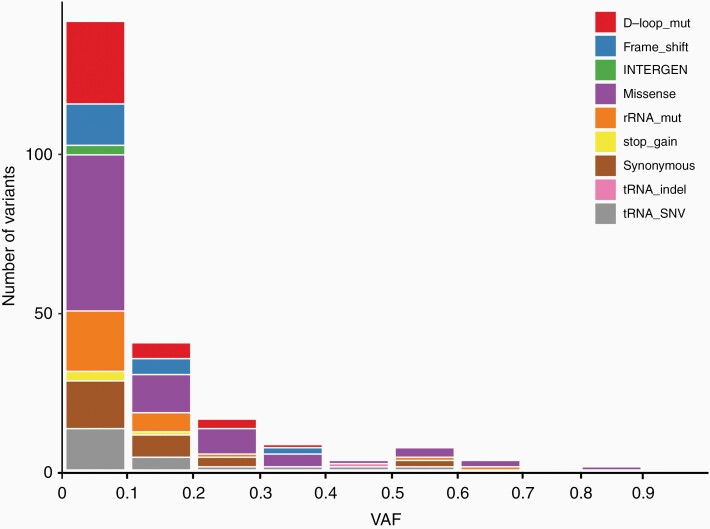
Number of mtDNA mutations at different variant allele frequencies (VAFs). mtDNA, mitochondrial DNA.

We have demonstrated the widespread and diverse nature of mtDNA mutations across various pediatric CNS tumor diagnoses (Figure 1). Missense mutations were most frequently observed overall across tumor types and such mutations appeared to cluster in genes encoding complex I. The average number of somatic mtDNA mutations per sample was less than 1 across all tumor diagnoses and the average heteroplasmy was also low, with less than 30% VAF across tumors of various diagnoses (Figure 5). This should not, however, discount the potential functional significance of mtDNA mutations in pediatric CNS tumors given that low heteroplasmy levels may reflect pooling artifact: it is possible that heteroplasmy levels are highly variable in different cells but obscured by bulk analysis. We have demonstrated the potential for mtDNA intratumoral heterogeneity with our single-cell sequencing analysis of 2 adult leukemia bone marrow samples in our previous study.^13^ In addition, we have previously shown that there are relatively fewer synonymous mutations compared to nonsynonymous mutations, especially in certain tumor types. For example, there are 19 nonsynonymous versus 2 synonymous mtDNA mutations in LGG, which is remarkably skewed compared to what would be expected due to random chance alone. This discrepancy is even more substantial than the average nonsynonymous versus synonymous ratio that we observed across pediatric tumor types (4.83) in our previous study, suggesting that these nonsynonymous mutations may be selected for and therefore more likely to be functionally significant.^13^

There are also notable differences between the various tumor subtypes, which again argues against mtDNA mutations being passenger or random events. The number of mtDNA mutations per sample, though low overall, ranged from lowest in the DNET group to the highest in meningioma. What is most striking to us is the observation that low-grade CNS tumors seem to harbor fewer mtDNA mutations than the high-grade and clinically more aggressive lesions, with the exception of AT/RT and possibly meningioma. Meningioma can be either a low-grade or high-grade lesion. It has been suggested that it could be more aggressive in the pediatric setting. While the surprisingly high number of mutations per sample might be explained by radiation exposure which is a known risk factor for this secondary lesion, most primary pediatric meningiomas are NF2 associated. It is, however, notable that an intrinsic difference between meningioma and other primary CNS tumors is its intrinsic histogenesis—it is a mesenchymal tumor and not a neuroepithelial tumor. Our findings are therefore consistent with a number of previous reports on the association of mtDNA mutations with the aggressiveness of a number of tumor types, including prostate cancer and fumarate hydratase-deficient renal cancer.^25,26^

A key finding of our analysis is that HGGs had a statistically significant higher number of mtDNA mutations per sample than LGGs (0.6 vs 0.27) (*P* = .003521), with almost twice as many missense mtDNA mutations per sample (0.24 vs 0.11), and higher average heteroplasmy levels (16% vs 10%). This is supported by a previous study with glioma cell lines.^27^ Even though it is unclear whether more mtDNA mutations contribute to the aggressiveness of the high-grade tumors or merely byproducts, such a clear correlation warrants further study for potentially therapeutic intervention.

### Mitochondrial Haplogroup Analysis Across CNS Tumors

Mitochondria are exclusively maternally inherited, allowing for assessment of maternal ancestry by interrogating the mitochondrial haplogroups based on common single nucleotide polymorphisms. For example, the L haplogroup is common in persons of African ancestry and the H haplogroup is common in persons of Eurasian ancestry. Some malignancies have been shown to have a strong association with certain ancestral backgrounds.^28–30^ This dataset shows a higher number of participants belonging to the H haplogroup. Instead of attributing this observation to higher risk for individuals of the H haplogroup, we believe that this is likely reflective of ascertainment bias and the common demographics of institutions which contributed to the CBTN database. Despite these limitations, however, we have shown that the different haplogroups appear to be equally represented across the various tumor types and no specific diagnosis appeared to be enriched for a particular mitochondrial haplogroup.

### mtDNA Mutations in Multiple Samples From the Same Patients

As noted in the results, WGS data from specimens obtained on the same day of surgery had shared and unique mtDNA mutations, which is consistent with intratumoral heterogeneity. In addition, in cases of multiple specimens over time, there is evidence of both shared mtDNA mutations and also mutations which were later lost or gained at the time of progression or recurrence. Differing mitochondrial mutation profiles may reflect tumor evolution possibly due to changes in the tumor microenvironment. We postulate that the emergence of new mutations in the setting of relapse or progression may be a mechanism in which mtDNA mutations play a role in tumor cell escape from treatment and/or adaptation to evolving tumor microenvironments. Several patients with multiple specimens may have had treatment-related secondary tumors (eg, a study subject with an initial diagnosis of MB followed by subsequent diagnosis of meningioma). This patient may have been treated with radiation therapy which led to the secondary meningioma. Similarly, patients with HGG after history of LGG likely had secondary HGG due to prior radiation therapy. Unfortunately, such detailed clinical information is unavailable and is a limitation of data-mining studies. Large institutional studies may provide more specific clinical data but would present a different set of limitations related to a low sample size, particularly in rare tumors.

### Functional Significance and Clinical Utility

Missense and LoF mutations are expected to result in protein changes and clustering of such mutations further suggests they may play a functionally significant role. Additionally, a number of such mutations were recurrent across different patients and different tumor types. For example, there were 4 frameshift mutations detected in patients with different histologic subtypes of CNS tumor (Supplementary Table 1). The mtDNA variants that we identified in gliosis samples demonstrate that nonmalignant conditions could also harbor mtDNA variants. However, those variants were not missense or loss-of-function mutations and may be consistent with the proliferative nature of gliosis. Further studies of gliosis are required to determine the significance of these findings. The possible functional significance of mtDNA mutations on tumorigenesis is further supported by the apparent clustering of mutations in complex I genes. This specific clustering of mtDNA mutations has also been previously reported in the setting of adult brain tumors, as well as in our pediatric pan cancer study.^13,31^ Interestingly, complex I has already been characterized as a possible “druggable target” with anticancer effects, further underscoring its potential oncogenic role. As an example, metformin has been shown to inhibit complex I and further work has demonstrated the use of metformin in human cancer cells leading to decreased proliferation.^32–34^ Another novel anticancer agent ONC201 has also been shown to cause breast cancer cell death in vitro by targeting mitochondria and leading to structural and functional mitochondrial impairments.^35,36^ The inhibition of cancer cell growth and survival by pharmacologically interfering with the oxidative phosphorylation (OXPHOS) complexes is compelling evidence for their roles in oncogenesis. Functional studies are needed, particularly to elucidate the metabolic functions of these mtDNA mutations and also their possible role in mitochondrial signaling and apoptosis. Considering our findings on the conservation of mtDNA mutations from diagnosis to disease progression, another potential clinical implication is the use of mtDNA mutations as a biomarker to study disease progression and response to treatment. Further studies utilizing serial tissue and/or liquid biopsies are needed to establish the utility of mtDNA mutations and copy numbers as biomarkers.

## Conclusion/Future Directions

In the present study, we characterized the mtDNA mutation profiles across pediatric CNS tumors. This data-mining study is limited due to the lack of detailed clinical information available from such publicly available datasets. There is now accumulating evidence that mtDNA mutations may play a role in the development and progression of pediatric brain tumors and functional studies in progress will help inform both the oncogenesis of these tumors and potential therapeutic pathways.

## Supplementary Material

vdab074_suppl_Supplementary_Figures

vdab074_suppl_Supplementary_Tables

## Notes

**Authorship Statement.** Project design: J.A.B. and X.G.; project oversight: J.A.B. and X.G.; data analysis: K.K., K.O., P.T., X.L., D.M., M.B., A.R., J.C., and D.O.; results interpretation: K.K., K.O., J.A.B., and X.G.; manuscript draft: K.K. and K.O.; manuscript review and edit: all.
