# Supplementary material for: The spectrum of mitochondrial DNA (mtDNA) mutations in pediatric CNS tumors
Source: Neurooncol Adv. 2021 Jun 2;3(1):vdab074. doi: 10.1093/noajnl/vdab074 (PMC8320689; doi:10.1093/noajnl/vdab074)

**Supplementary Figure 1: Histologic diagnoses of patients in the data set**


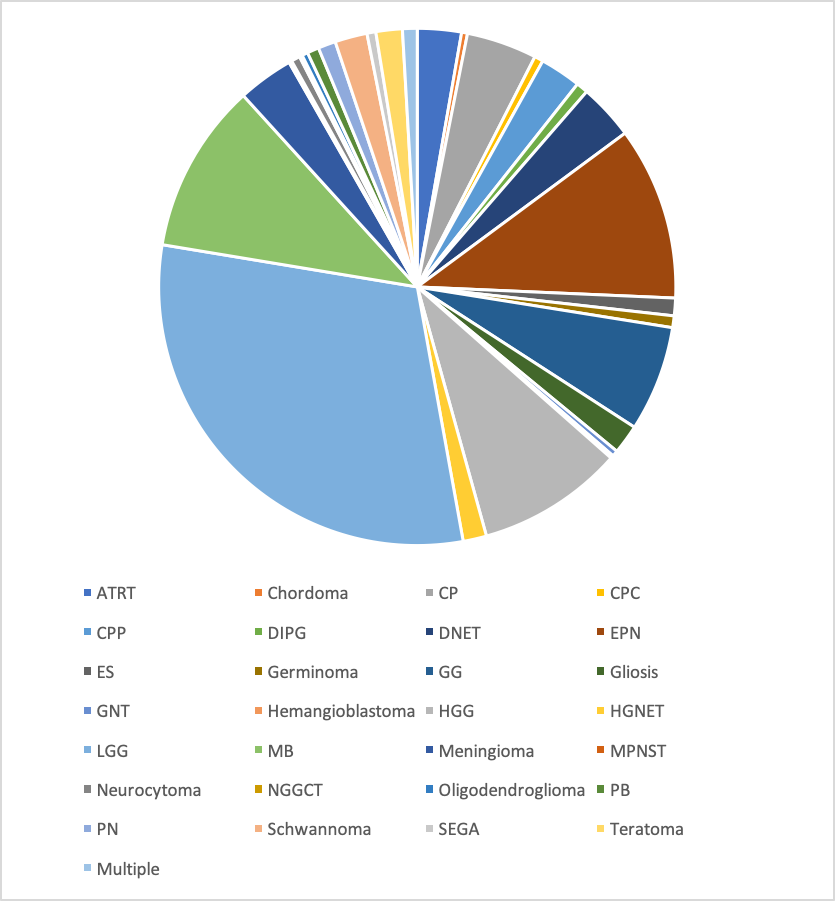


**Supplementary Figure 2: Mitochondrial haplogroups for patients in the study.**

The number of cases in each haplogroup is included in parentheses.


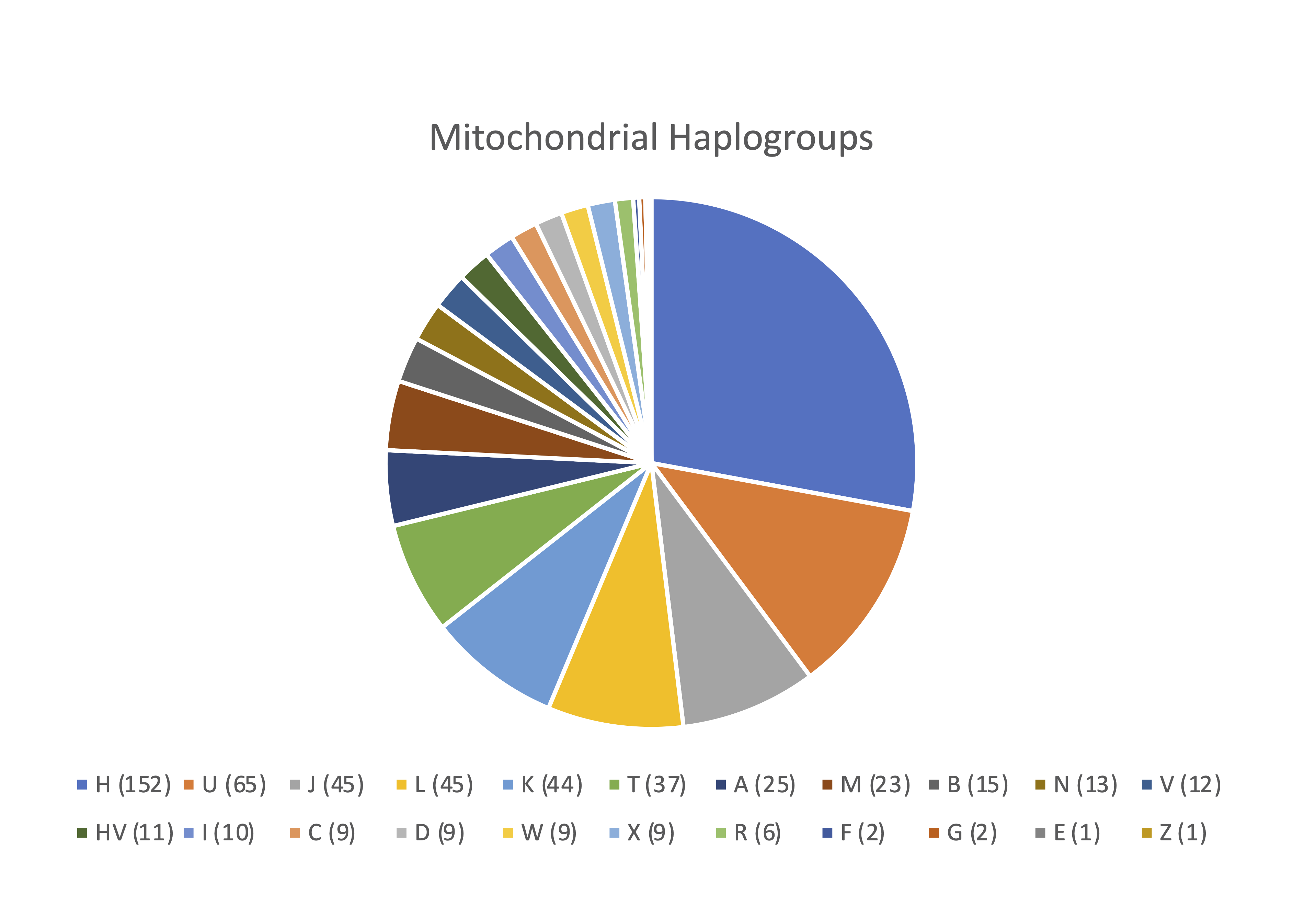

Supplement: vdab074_suppl_Supplementary_Figures [file vdab074_suppl_supplementary_figures.docx]
